# Intratracheal Delivery of a Phospholamban Decoy Peptide Attenuates Cardiac Damage Following Myocardial Infarction

**DOI:** 10.3390/ijms26062649

**Published:** 2025-03-14

**Authors:** Taewon Kook, Mi-Young Lee, Tae Hwan Kwak, Dongtak Jeong, Doo Sun Sim, Myung Ho Jeong, Youngkeun Ahn, Hyun Kook, Woo Jin Park, Seung Pil Jang

**Affiliations:** 1 School of Life Sciences, Gwangju Institute of Science and Technology, Gwangju 61005, Republic of Korea; 2 BethphaGen, S3-203, Gwangju 61005, Republic of Korea; 3 Department of Medicinal & Life Science, College of Science and Convergence Technology, Hanyang University-ERICA, Ansan 15588, Republic of Korea; cooljdt@hanyang.ac.kr; 4 Department of Cardiology, Cardiovascular Center, Chonnam National University Hospital, Gwangju 61469, Republic of Korea; 5 Department of Pharmacology, Chonnam National University Medical School, Hwasun 58128, Republic of Korea; 6 Center for Gene and Cell Therapy, Korea Research Institute of Bioscience and Biotechnology, Daejeon 34141, Republic of Korea

**Keywords:** intratracheal delivery, myocardial infarction, decoy peptide, phospholamban, protein phosphatase 1, PP1cα, PPP1R3A

## Abstract

Heart failure (HF) remains a major cause of mortality worldwide. While novel approaches, including gene and cell therapies, show promise, efficient delivery methods for such biologics to the heart are critically needed. One emerging strategy is lung-to-heart delivery using nanoparticle (NP)-encapsulated biologics. This study examines the efficiency of delivering a therapeutic peptide conjugated to a cell-penetrating peptide (CPP) to the heart via the lung-to-heart route through intratracheal (IT) injection in mice. The CPP, a tandem repeat of NP2 (dNP2) derived from the human novel LZAP-binding protein (NLBP), facilitates intracellular delivery of the therapeutic payload. The therapeutic peptide, SE, is a decoy peptide designed to inhibit protein phosphatase 1 (PP1)-mediated dephosphorylation of phospholamban (PLN). Our results demonstrated that IT injection of dNP2-SE facilitated efficient delivery to the heart, with peak accumulation at 3 h post-injection. The administration of dNP2-SE significantly ameliorated morphological and functional deterioration of the heart under myocardial infarction. At the molecular level, dNP2-SE effectively prevented PLN dephosphorylation in the heart. Immunoprecipitation experiments further revealed that dNP2-SE binds strongly to PP1 and disrupts its interaction with PLN. Collectively, our findings suggest that lung-to-heart delivery of a CPP-conjugated therapeutic peptide, dNP2-SE, represents a promising approach for the treatment of HF.

## 1. Introduction

Heart failure (HF) remains a leading cause of mortality, morbidity, and diminished quality of life worldwide. Despite advances in traditional medications, the prevalence of HF continues to rise [1,2,3]. To address this unmet medical need, novel therapeutic strategies involving biologics, such as viral vectors, cells, peptides, and nucleic acids, are being actively developed [4,5,6,7]. These biologics can be delivered to the heart through various routes, including intramuscular (IM), intravenous (IV), intraperitoneal (IP), and intracoronary (IC) artery administration. However, these delivery methods are often constrained by challenges such as variable absorption, non-specific biodistribution, and the requirement for frequent hospital visits.

Recent studies have proposed lung-to-heart delivery via intratracheal (IT) injection or inhalation as a promising strategy for delivering biologics to the heart [8,9,10]. Nanoparticles (NPs) with diameters less than 100 nm have been shown to efficiently traverse the lungs to reach the heart. The inhaled biologics dissolve in lung fluid, enabling the release of therapeutic molecules, which then translocate across the alveolocapillary membrane into the cardiopulmonary circulation. Therapeutic biologics, such as miRNAs and peptides encapsulated in NPs, delivered via the lung-to-heart route have demonstrated the ability to ameliorate cardiac dysfunctions in animal models of HF [11,12]. Despite these promising findings, several challenges hinder the clinical application of NP-encapsulated biologics. NPs themselves carry potential toxicity risks, highlighting the need for long-term safety evaluations. Additionally, manufacturing NPs presents significant obstacles. Achieving precise control over the particle size, surface characteristics, and drug release profiles introduces complexity and increases production costs, posing barriers to large-scale implementation.

In this study, we aimed to evaluate the feasibility of delivering a naked peptide fused with a cell-penetrating peptide (CPP) to the heart via the lung-to-heart route without encapsulation in NPs. CPPs, which are the peptide sequences enriched in arginine and lysine residues, facilitate the translocation of hydrophilic macromolecules such as proteins and peptides across cellular membranes [13,14]. The specific CPP used in this study is a tandem repeat of NP2 (dNP2), a sequence derived from the human novel LZAP-binding protein (NLBP). Compared to TAT, the most widely used CPP originated from HIV-1, dNP2 has demonstrated superior translocation capabilities, enabling it to cross vascular endothelial cells and reach parenchymal cells effectively. Furthermore, the human protein-originated dNP2 exhibits significantly lower toxicity than TAT, making it more favorable for clinical applications [15,16,17].

The therapeutic peptide used in this study is a 9-mer peptide, RAE^16^TIEMPQ, derived from the human phospholamban (PLN) protein sequence. This peptide includes a serine (S)-to-glutamate (E) substitution at position 16, thus referred to as SE [18,19]. This S-to-E substitution mimics the phosphorylation of PLN at S^16^. Sarcoplasmic reticulum Ca^2+^-ATPase (SERCA2a) plays a crucial role in Ca^2+^ handling in cardiomyocytes [20,21,22]. PLN, an endogenous inhibitor of SERCA2a, has its inhibitory activity enhanced via dephosphorylation at S^16^ by protein phosphatase 1 (PP1) [23,24]. Therefore, inhibiting PP1-mediated dephosphorylation of PLN at S^16^ represents an efficient strategy for the restoration of reduced SERCA2a activity in failing hearts [25]. We hypothesized that SE could prevent the dephosphorylation of PLN at S^16^ by acting as a decoy substrate for PP1. Previous studies demonstrated that TAT-SE effectively restored impaired Ca^2+^ handling and contractility of cardiomyocytes both in vitro and in vivo under various pathological conditions.

In this study, we demonstrated that dNP2-SE can be efficiently delivered to the heart via the lung-to-heart route and effectively prevents morphological, functional, and molecular deteriorations of the heart in a mouse model of myocardial infarction (MI). As hypothesized, we also observed that dNP2-SE disrupts the direct interaction between PP1 and PLN.

## 2. Results

### 2.1. Delivery of dNP2-SE to the Heart via IT Injection

**Figure 1:**
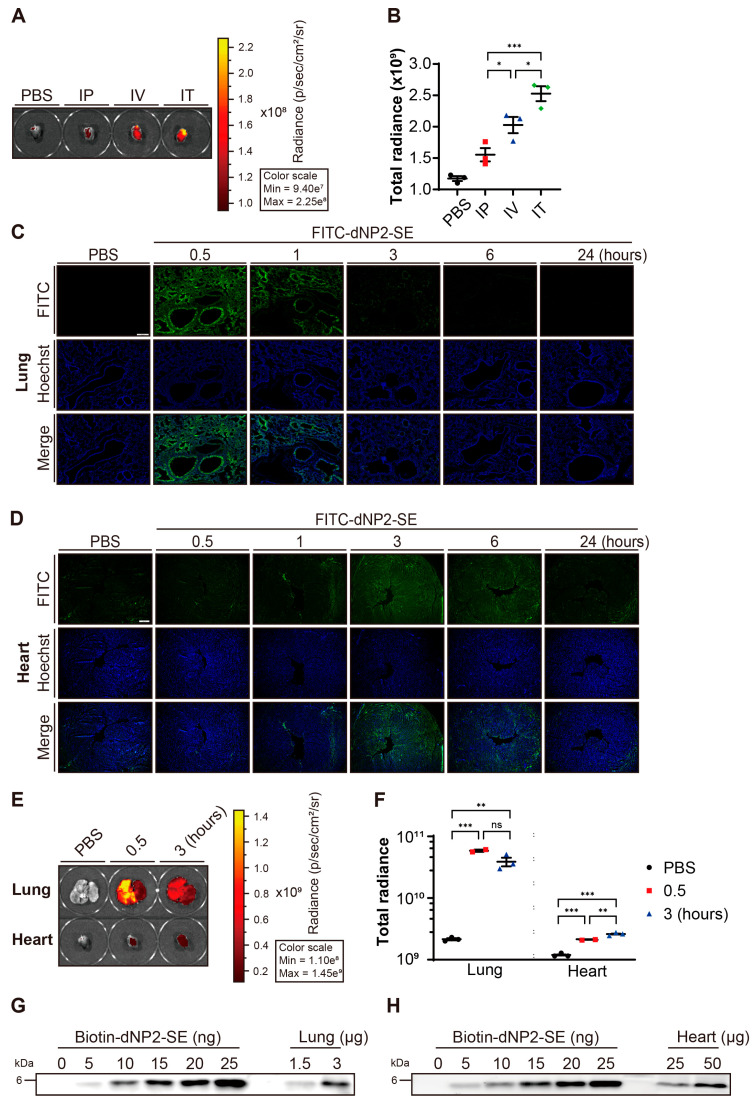
Delivery of dNP2-SE to the heart via IT injection. (**A**) A total of 200 nmoles of FITC-dNP2-SE was delivered via IP, IV, and IT injection. PBS was injected as the control group. Hearts were harvested after 3 h and fluorescence was measured using an in vivo imaging system. (**B**) Fluorescence intensities in the Heart were quantified and presented as a graph. n = 3 per group; one-way ANOVA; * *p* < 0.05, *** *p* < 0.001. Bar represents the mean ± SEM. (**C**,**D**) A total of 200 nmoles of FITC-dNP2-SE was delivered via IT injection. The lung and heart tissues were obtained at 0.5, 1, 3, 6, and 24 h post-injection, sectioned, and stained with Hoechst 33342 dye. The fluorescences from FITC (green) and Hoechst 33342 (blue) were observed using a fluorescent slide scanner. (**E**) A total of 200 nmoles of FITC-dNP2-SE was delivered via IT injection. Lungs and hearts were harvested after 0.5 and 3 h post-injection, and fluorescence was measured using an in vivo imaging system. (**F**) Fluorescence intensities in the lungs and hearts were quantified and presented as a graph. n = 3 per group; one-way ANOVA; ** *p* < 0.01, *** *p* < 0.001. Bar represents the mean ± SEM. (**G**,**H**) A total of 200 nmoles of biotin-dNP2-SE was delivered via IT injection. Lungs and hearts were harvested 3 h post-injection. Protein lysates were prepared and analyzed by western blotting. To generate a standard curve, 5~25 ng of biotin-dNP2-SE were loaded. The amounts of biotin-dNP2-SE in lungs and hearts were estimated by interpolation on the standard curve.

We evaluated the efficiency of delivering the dNP2-SE peptide to the heart via different administration routes: IP, IV, and IT injections. To assess delivery efficiency, FITC-labeled dNP2-SE (FITC-dNP2-SE) was used, and hearts were collected at 3 h post-injection. Among the tested routes, IT injection resulted in the highest peptide delivery to the heart (Figure 1A,B).

After delivering FITC-dNP2-SE via IT injection, the lung and the heart were collected at various time points (0.5, 1, 3, 6, and 24 h), sectioned, and analyzed using a fluorescent slide scanner. In the lung, fluorescence peaked at the earliest time point (0.5 h) and gradually decreased over time (Figure 1C). In contrast, fluorescence in the heart increased more slowly, peaking at 3 h post-injection before gradually declining (Figure 1D). Direct imaging corroborated these findings, showing peak fluorescence at 0.5 h post-IT injection in the lung and at 3 h post-IT injection in the heart (Figure 1E,F). To further quantify delivery efficacy, 200 nmoles (816 µg) of biotin-labeled peptide (biotin-dNP2-SE) was administered via IT injection. Lung and heart tissues were collected and subjected to semi-quantitative western blotting. Approximately 8.8 nmoles (36 µg) of the injected peptide were detected in the lung, while around 0.6 nmoles (2.6 µg) of the peptide were detected in the heart (Figure 1G,H, Supplementary Figure S1). These results indicate that approximately 4.4% and 0.3% of the IT-injected peptides reached the lung and the heart, respectively, at 3 h post-injection.

### 2.2. Delivery of dNP2-SE via IT Injection Exerts Cardio-Protective Effects upon MI

**Figure 2:**
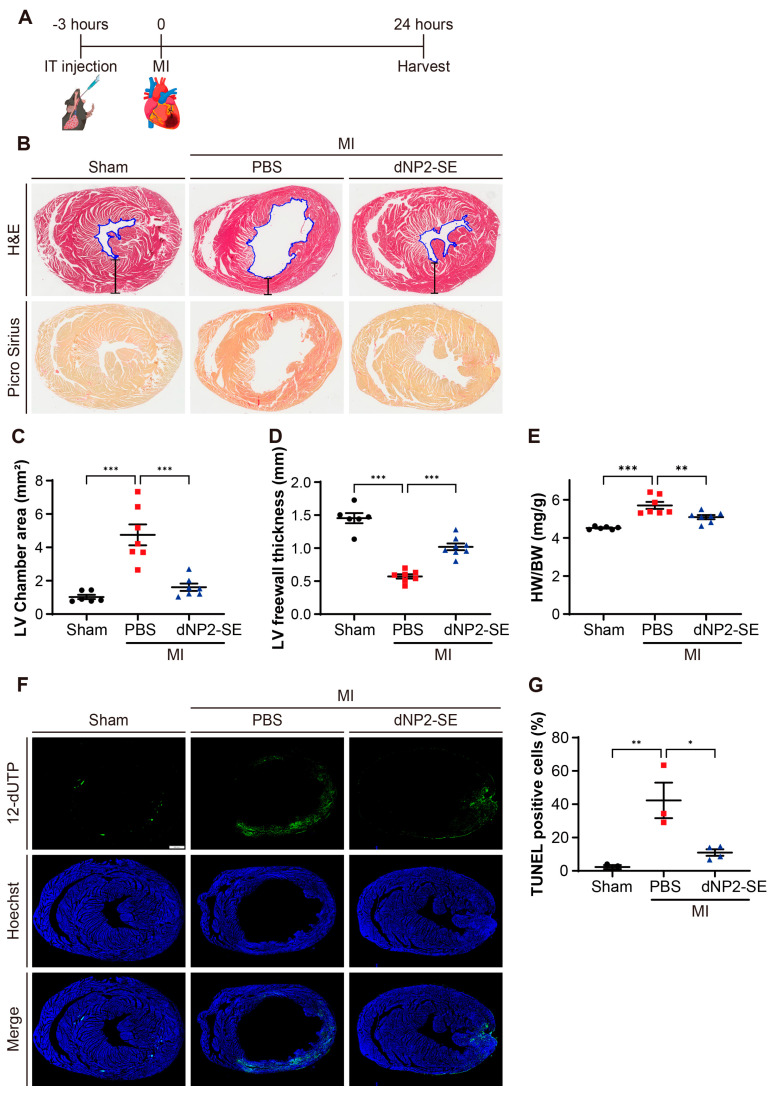
Delivery of dNP2-SE via IT injection exerts cardio-protective effects upon MI. (**A**) Experimental scheme. A total of 200 nmol dNP2-SE or PBS was delivered via IT injection to adult (9-week-old) C57/Bl6 mice. MI was induced by LAD ligation 3 h post-injection. Hearts were harvested and analyzed 24 h post-MI. (**B**) Hearts were sectioned and stained with hematoxylin and eosin (H&E) or Picro Sirius Red. Representative images are shown. The LV chamber area was traced (shown in blue lines) and quantitated. The thickness of the LV free wall was also measured (shown in straight black lines). (**C**,**D**) LV chamber area and LV free wall thickness are presented as a graph. n = 6~7 per group; one-way ANOVA; *** *p* < 0.001. Bar represents the mean ±SEM. (**E**) HW/BW ratio was measured and shown. n = 6~7 per group; one-way ANOVA; ** *p* < 0.01, *** *p* < 0.001. Bar represents the mean ±SEM. (**F**) TUNEL staining of the heart sections was performed using the DeadEnd Fluorometric TUNEL System. Apoptotic DNA fragmentation was labeled with fluorescein-12-dUTP (green). Nuclei were stained with Hoechst 33342 (blue). Representative images are shown. (**G**) TUNEL-positive cells and the nucleus in the LV free wall areas were counted, and the ratio of TUNEL-positive cells is shown. n = 3~4 per group; one-way ANOVA; * *p* < 0.05, ** *p* < 0.01. Bar represents the mean ± SEM.

A dose of 200 nmoles of dNP2-SE was delivered via IT injection at 3 h prior to inducing MI in mice (Figure 2A). Staining of heart sections with Picro Sirius revealed significant collagen deposition in PBS-injected hearts under MI, which was markedly reduced by IT delivery of dNP2-SE (Figure 2B). The left ventricular (LV) chamber area, outlined with blue traces in Figure 2B, was prominently enlarged in PBS-injected hearts but remained normal in dNP2-SE-injected hearts under MI (Figure 2C). Similarly, the LV free wall thickness, marked by bracketed lines in Figure 2B, was greatly reduced in PBS-injected hearts under MI but was significantly preserved in dNP2-SE-injected hearts (Figure 2D). The heart weight-to-body weight ratio, which was significantly increased by MI in PBS-injected hearts, was normalized in dNP2-SE-injected hearts (Figure 2E). TUNEL staining of heart sections revealed a significant increase in apoptotic cell populations in PBS-injected hearts under MI, whereas this increase was effectively prevented in dNP2-SE-injected hearts (Figure 2F,G). These findings collectively indicate that IT injection-mediated delivery of dNP2-SE significantly mitigated fibrosis, morphological deterioration, and apoptosis in the heart under MI.

**Figure 3:**
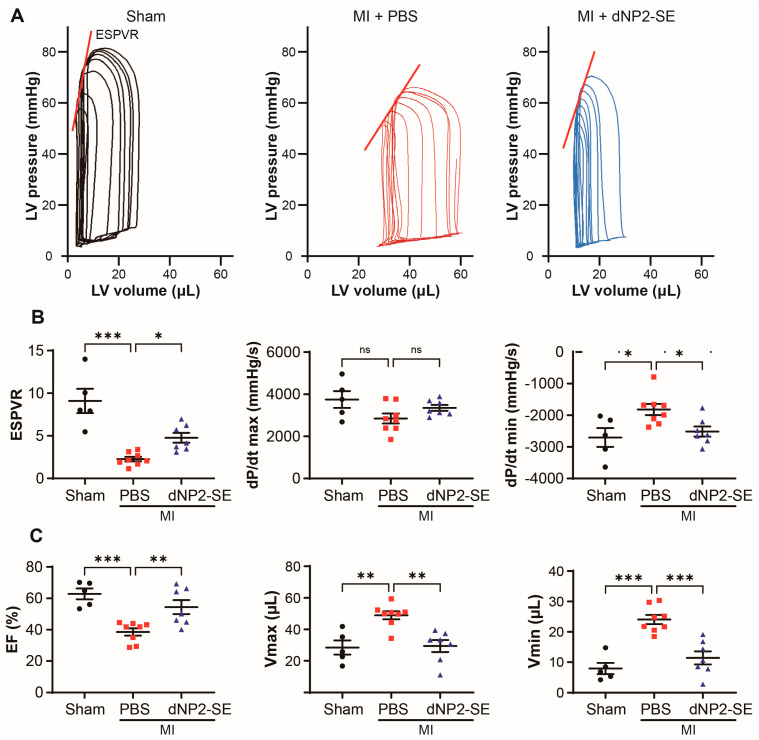
Delivery of dNP2-SE via IT injection preserves cardiac contractility upon MI. Hemodynamic analyses were performed 24 h post-MI to measure cardiac contractility. (**A**) Representative PV loops are shown. (**B**) End-systolic pressure–volume relationship (ESPVR), dp/dt max (mmHg), and dp/dt min (mmHg) are shown. (**C**) Ejection fraction (EF), LV end-diastolic volume (Vmax), and LV end-systolic volume (Vmin) are shown. n = 5~8 per group; one-way ANOVA; * *p* < 0.05, ** *p* < 0.01, *** *p* < 0.001. Bar represents the mean ± SEM.

Hemodynamic measurements revealed that PBS-injected hearts under MI exhibited pressure–volume (PV) loop patterns typically seen in hearts with reduced systolic and diastolic function. In contrast, the PV loop pattern of dNP2-SE-injected hearts under MI was indistinguishable from that of sham-operated hearts (Figure 3A). The end-systolic pressure–volume ratio (ESPVR) and dP/dt max were significantly reduced, while dP/dt min was markedly increased in PBS-injected hearts under MI. These functional impairments were significantly normalized in dNP2-SE-injected hearts (Figure 3B). Additionally, the reduced ejection fraction (EF) and increased Vmax and Vmin observed in PBS-injected hearts were significantly ameliorated in dNP2-SE-injected hearts under MI (Figure 3C). These findings demonstrate that IT injection-mediated delivery of dNP2-SE effectively prevents cardiac dysfunction following MI.

### 2.3. Delivery of dNP2-SE via IT Injection Prevents Molecular Abnormalities upon MI

**Figure 4:**
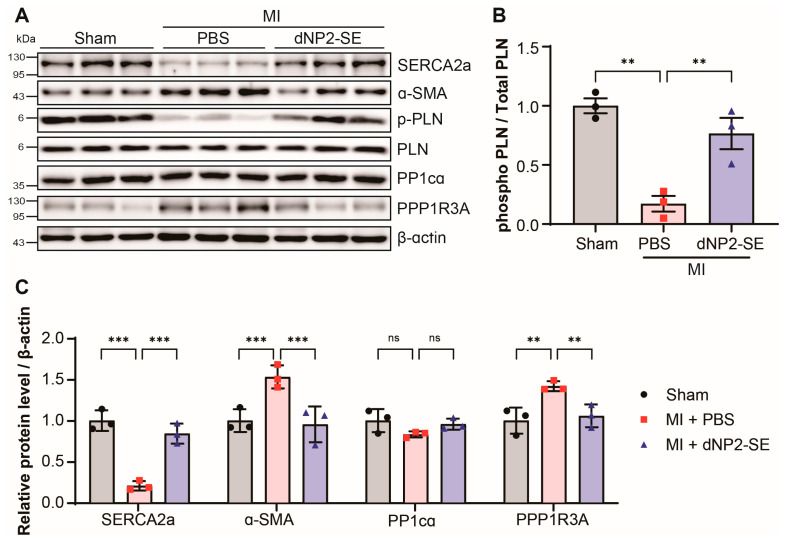
Delivery of dNP2-SE via IT injection prevents molecular abnormalities upon MI. (**A**) Protein lysates obtained from the hearts shown in Figure 2 and Figure 3 were subjected to western blotting. Antibodies against SERCA2a, α-SMA, phospho-PLN, PLN, PP1cα, and PPP1R3A were used. β-actin was used as a loading control. (**B**) The ratio of phosphorylated PLN over PLN is shown. (**C**) The western blots were scanned and quantitated. n = 3 per group; one-way ANOVA; ** *p* < 0.01, *** *p* < 0.001. Bar represents the mean ± SEM.

Heart tissues obtained from the experiments in Figure 2 and Figure 3 were subjected to western blotting to evaluate the effects of dNP2-SE at the molecular level (Figure 4A). The PBS-injected hearts following MI exhibited a reduced expression of SERCA2a and an increased level of α-smooth muscle actin (α-SMA), both hallmarks of failing hearts. These molecular changes were attenuated by IT-mediated delivery of dNP2-SE (Figure 4A,C). Additionally, the phosphorylation of PLN at S^16^ (p-PLN) was significantly reduced in PBS-injected hearts under MI but was fully preserved in dNP2-SE-treated hearts (Figure 4A,B). This observation aligns with the hypothesis that SE acts as a decoy substrate for PP1, inhibiting the dephosphorylation of PLN at S^16^. The expression level of PP1cα, the α catalytic subunit of PP1, remained unchanged. However, the expression of PPP1R3A, a regulatory subunit of PP1 known to recruit substrates such as PLN to the PP1 complex, was significantly increased following MI. This increase in PPP1R3A expression was prevented by dNP2-SE treatment (Figure 4A,C), which may account for the reduced dephosphorylation of PLN observed with dNP2-SE. These findings demonstrate that IT-mediated delivery of dNP2-SE effectively inhibits PLN de-phosphorylation and mitigates associated molecular abnormalities following MI.

### 2.4. Inhibitory Mechanism of dNP2-SE on the PP1cα-PPP1R3A-PLN Complex

**Figure 5:**
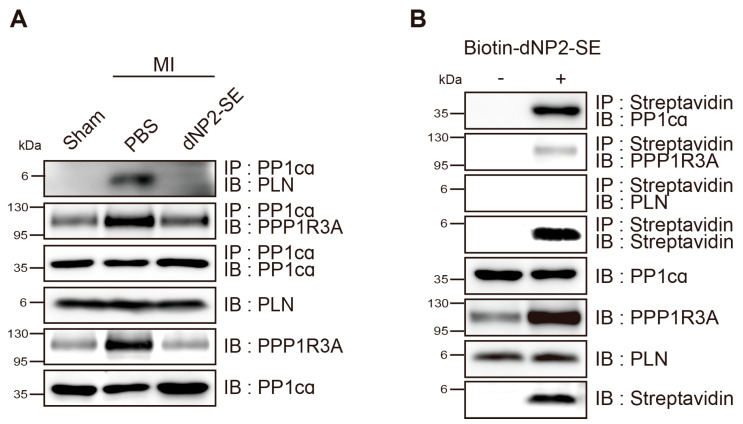
Inhibitory mechanism of dNP2-SE on the PP1cα-PPP1R3A-PLN complex. (**A**) Protein lysates shown in Figure 4 were subjected to immunoprecipitation with anti-PP1cα antibodies and blotted with antibodies against PLN, PPP1R3A, and PP1cα (upper three rows). Inputs were also blotted with antibodies against PLN, PPP1R3A, and PP1cα (lower three rows). (**B**) Biotin-dNP2-SE was delivered to the normal heart via IT injection. Heart lysates were obtained, precipitated with streptavidin, and blotted with PP1cα, PPP1R3A, and PLN. Inputs were also blotted.

Protein lysates were prepared from the heart tissues shown in Figure 2 and Figure 3 and then subjected to immunoprecipitation analysis. Notable physical interactions between PP1c*α* and PLN were observed in PBS-injected hearts following MI, which were completely abolished in dNP2-SE-injected hearts. However, the interactions between PP1c*α* and PPP1R3A were not altered by dNP2-SE treatment (Figure 5A). Biotin-dNP2-SE was delivered to the heart via IT injection, as described in Figure 1H, and protein lysates were prepared. Using streptavidin, the peptide was precipitated, and the precipitated materials were analyzed by western blotting. The results demonstrated that dNP2-SE strongly binds to PP1c*α* but only weakly interacts with PPP1R3A (Figure 5B). These findings collectively support our hypothesis that SE acts as a decoy substrate for PP1, thereby preventing the dephosphorylation of PLN by PP1.

## 3. Discussion

Recent studies suggest the potential of lung-to-heart delivery as an alternative strategy for targeting the heart. IT injection provides direct access to the pulmonary and coronary circulations, facilitating the delivery of NPs or NP-encapsulated drugs to the heart [11,26,27]. Compared to traditional IP or IV routes, IT injection is less invasive and offers higher efficiency for heart-targeted delivery. We aimed to evaluate the effectiveness of naked peptides conjugated to a CPP compared to NP-encapsulated formulations for lung-to-heart delivery. First, we compared TAT and dNP2 as CPP moieties. TAT, derived from HIV-1, is widely used for various in vivo and in vitro experiments [15]. However, dNP2 presents an attractive alternative for two reasons: (1) it is derived from a human protein, making it potentially safer for long-term use with reduced immunogenicity and toxicity compared to the virus-derived TAT, and (2) dNP2 efficiently penetrates vascular endothelial cells, facilitating translocation to the parenchyma. When delivered directly into circulation, TAT-linked proteins tend to remain in the bloodstream, while dNP2-linked proteins efficiently exit the circulation and translocate to the parenchyma of various tissues. To compare the delivery efficiency, FITC-labeled TAT-SE and dNP2-SE were prepared and administered via IT injection. Hearts were harvested at 3 h post-injection, and significantly higher fluorescence was observed in the hearts of mice treated with FITC-dNP2-SE compared to FITC-TAT-SE (Supplementary Figure S2). Based on these findings, we used dNP2-SE throughout this study. Our data indicate that dNP2 is an efficient CPP for IT-mediated heart delivery. However, further studies are needed to evaluate other human protein-derived CPPs for this purpose.

**Figure 6:**
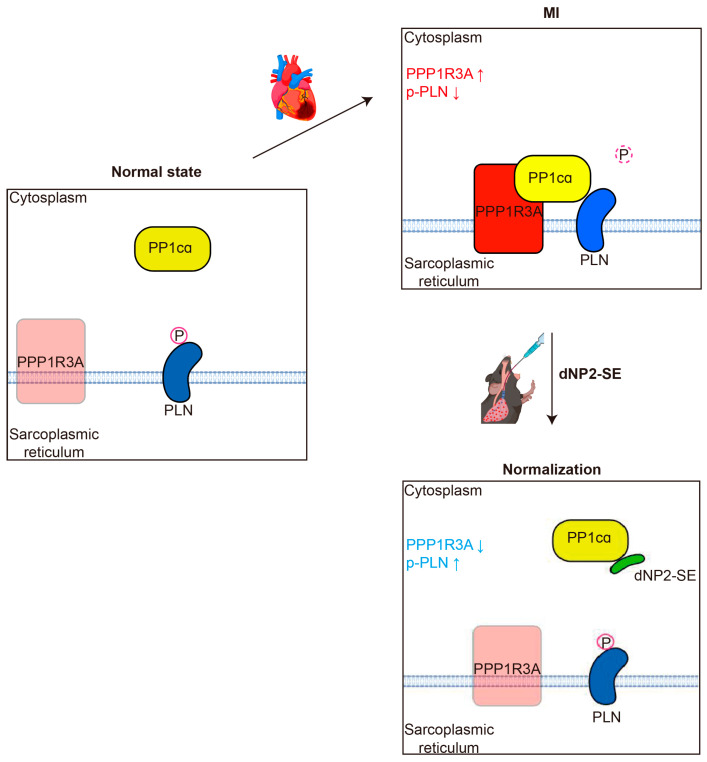
Proposed role of dNP2-SE on PLN dephosphorylation. In normal hearts, phosphorylation of PLN remains high. Upon MI, the elevated expression level of PPP1R3A results in a higher level of the PP1 holoenzyme, PP1cα-PPP1R3A leading to increased de-phosphorylation of PLN. dNP2-SE interferes in the direct association of PP1cα and PLN by serving as a decoy peptide, which leads to a decreased level of PPP1R3A via an unknown mechanism.

Reduced expression or activity of SERCA2a, a key regulator of Ca^2+^ handling in cardiomyocytes, is associated with HF [21,28]. Restoring SERCA2a levels through gene delivery modalities has been shown to effectively ameliorate HF phenotypes in various animal models and humans [29]. PLN is an endogenous inhibitor of SERCA2a, and its inhibitory activity is enhanced by dephosphorylation at S^16^ and T^17^ by PP1 [30,31]. Consequently, inhibition of PP1-mediated PLN dephosphorylation has emerged as a viable therapeutic target for treating HF [32,33]. Previously, we hypothesized that a 9-mer peptide, referred to as SE, could mimic the phosphorylated PLN, thus serving as a decoy substrate for PP1. Furthermore, we demonstrated that TAT-SE effectively prevents PLN dephosphorylation both in vitro and in vivo [18,19]. For the first time, the current study shows that: (1) SE directly binds to the catalytic domain of PP1 (PP1cα), and (2) SE disrupts the direct interaction between PP1 and PLN. These results are consistent with our original hypothesis. Interestingly, we observed that the level of PPP1R3A, a regulatory subunit of PP1, is elevated following MI but is normalized by SE treatment. It is plausible that PPP1R3A becomes prone to protein degradation when the formation of the PP1cα- PPP1R3A-PLN complex is disrupted, further enhancing the interaction between PP1cα and PLN (Figure 6). Further studies will be needed to explore the association between protein degradation and the regulation of PPP1R3A.

Limitations: In this study, we did not directly compare the efficacy of the CPP-linked peptide with that of the NP-encapsulated peptide in lung-to-heart delivery. Additionally, we did not determine the lowest effective dose of dNP2-SE. Further studies involving more precise comparisons and dose-deescalation experiments are warranted.

Despite these limitations, our findings clearly demonstrate that the naked peptide dNP2-SE, delivered via IT injection, represents a promising therapeutic strategy for HF.

## 4. Materials and Methods

### 4.1. Design and Synthesis of dNP2-SE

A 9-mer peptide, SE (RAE^16^TIEMPQ), is derived from the human PLN protein sequence, with a serine (S)-to-glutamate (E) substitution at position 16. This S-to-E substitution is intended to mimic the phosphorylation of PLN at S^16^. The decoy peptide, dNP2-SE, was designed by placing the SE peptide sequence at the C-terminus of a CPP, dNP2, (KIKKVKKKGRKGSKIKKVKKKGRKG). The dNP2-SE peptide was synthesized with additional modifications, N-terminal acetylation, and C-terminal amidation. To facilitate detection and measurement, FITC- or biotin-conjugated dNP2-SE was also synthesized. All the peptides were produced by AnyGen (Gwangju, Korea) with a purity of >95% and dissolved in PBS to a concentration of 4 nmol/µL [18].

### 4.2. Animals

Eight-week-old C57BL/6 mice were purchased from Daehan Biolink (Eumseong, Korea) and housed in an animal facility for a one-week adaptation period. The mice were fed a normal chow diet. IT injection and surgical procedures were conducted under anesthesia, administered via IP injection of xylazine (5–12 mg/kg) and ketamine (70–100 mg/kg).

### 4.3. IT Injection

Incisions were made with scissors in the skin above the sternal notch. The muscles covering the trachea were separated using a retractor. A microsprayer (KN-34700, Natume, Japan) was inserted into the trachea, and 50 µL (200 nmol) of the peptide solution was injected by rapidly pressing the plunger.

### 4.4. Fluorescent Slide Scanning

Harvested lung and heart tissues were fixed in 4% paraformaldehyde for 24 h at 4 °C and then incubated in 30% sucrose for 48 h at 4 °C as a cryoprotectant. The tissues were embedded in an OCT compound (3801480, Leica, Düsseldorf, Germany). Frozen tissue blocks were sectioned at 20 µm and 8 µm thickness for lung and heart, respectively, using a cryostat microtome (HM525NX, Thermo Scientific, Waltham, MA, USA). The sections were washed with PBS and counterstained with Hoechst 33342 (Invitrogen, Waltham, MA, USA) to visualize cell nuclei. The slides were then mounted with an anti-fade mounting medium and examined under a fluorescent slide scanner (VS200, OLYMPUS, Tokyo, Japan).

### 4.5. Measurement of Peptide in the Lung and Heart

Two hundred nmol of FITC-conjugated dNP2-SE peptide was injected via IP, IV, and IT routes. The lung and heart were harvested at the desired time point and fixed in 4% paraformaldehyde for 24 h. Fluorescence of the samples was measured using the IVIS^®^ imaging system (PerkinElmer, Waltham, MA, USA).

### 4.6. Western Blot

Heart lysates were obtained by adding RIPA buffer (1% IGEPAL, 25 mM Tris, pH 8.0, 15 mM NaCl, 5 mM EDTA, 2 mM NaF, 0.5% sodium deoxycholate, 0.1% SDS, 10 mM PMSF, and a protease inhibitor cocktail) to the tissue, followed by sonication. Proteins were separated by SDS-PAGE using a 10% acrylamide gel and PeptiGel based on their molecular weights. After separation, the protein-loaded gel was transferred to polyvinylidene fluoride (PVDF) membranes (Merck, Darmstadt, Germany) with a 0.45 µm pore size. The membranes were then blocked with 5% (*w*/*v*) skim milk for 30 min and washed with TBS-T. Subsequently, the membranes were incubated with the desired antibodies overnight at 4 °C, washed, and incubated for 1 h at room temperature with horseradish peroxidase-conjugated secondary antibodies. Detection was performed using the EZ-Western Lumi-Pico Kit (DG-WP250, DoGenBio, Seoul, Republic of Korea). Chemiluminescence signals were detected using the Amersham™ ImageQuant™ 800 (Cytiva, Dreieich, Germany).

The primary antibodies used in this study were as follows: anti-Streptavidin (ab7406, Abcam, Cambridge, UK), anti-PLN pSER16 (A010-12AP, Badrilla, Leeds, UK), anti-PLN (A010-14, Badrilla, UK), anti-PP1cα (A24288, Abclonal, Woburn, MA, USA), anti-PPP1R3A (sc-398425, Santa Cruz, CA, USA), anti-SERCA2a (sc-53010, Santa Cruz, CA, USA), anti-α-smooth muscle actin (SAB5700835; Sigma-Aldrich, Burlington, MA, USA), and anti-β-actin (sc-47778, Santa Cruz, USA).

### 4.7. Myocardial Infarction (MI)

The left side of the chest was shaved, and an intubation tube was inserted into the trachea. An incision was made in the 4th intercostal space, and the muscles were separated with scissors. The pericardium was gently removed. MI was induced by ligation of the left anterior descending (LAD) artery using a 7–0 black silk suture. The lower region of the artery turned pale immediately after ligation. The chest was sutured with 4–0 black silk. The same surgical procedure, without LAD ligation, was performed for the sham operation.

### 4.8. Histology and TUNEL Assays

Harvested heart tissues were fixed in 4% PFA for 24 h at 4 °C and then washed with tap water. After discarding the tap water, the tissues were sequentially incubated in 70%, 80%, 95%, and 100% ethanol for 1 h each to dehydrate. Following the ethanol incubations, the tissues were incubated in xylene for 1 h and 20 min and then embedded in paraffin at 60 °C for 4 h. Paraffin-embedded tissues were sliced into 8 µm sections. After rehydration, the sections were used for histology as follows:

H&E staining: The sections were incubated in hematoxylin (26041-06, Electron Microscopy Science, Hatfield, PA, USA) for 30 s to stain the nuclei, followed by a 10 min wash in running tap water to remove excess stain. Subsequently, the sections were incubated in eosin (EOYA-05-OT-1L, Biognost, Zagreb, Croatia) for 2 min to stain the cytoplasm.

Picro Sirius staining: The sections were incubated for 60 min in Picro Sirius Red solution (ab150681, Abcam, Waltham, MA, USA), rinsed twice with 0.5% acetic acid, and then rinsed with absolute alcohol. H&E and Picro Sirius-stained slides were observed using a slide scanner (Aperio CS2, Leica, Germany).

The TUNEL assay was performed following the manufacturer’s instructions (DeadEnd Fluorometric TUNEL System, Promega, San Luis Obispo, CA, USA). After rehydration, the sections were incubated with Proteinase K (20 µg/mL) for 15 min at room temperature to permeabilize the tissues. Following a PBS wash, the sections were treated with a TUNEL reaction mixture containing terminal deoxynucleotidyl transferase (TdT) and fluorescein-dUTP. The reaction was carried out in a humidified chamber at 37 °C for 1 h. After the reaction, the sections were stained with Hoechst 33342 and examined under a fluorescent slide scanner (VS200, OLYMPUS, Japan). Apoptotic signals and nuclear signals in the LV region were counted using cellSens TruAI 4.3 software (OLYMPUS, Tokyo, Japan), and the percentage of TUNEL-positive cells was calculated.

### 4.9. Hemodynamics Analysis

An incision was made in the abdominal wall to expose the abdominal cavity. The diaphragm was carefully separated from the ribs to avoid bleeding during the incision procedures. The xiphoid process was lifted, and the diaphragm was pulled down and fixed to secure the site. The pericardium was then removed. A small puncture was made in the left ventricle using an insulin syringe, and a probe was inserted through the puncture (AD instruments, Dunedin, New Zealand). Data analysis was performed using LabChart 8 (AD instruments, New Zealand).

### 4.10. Immunoprecipitation

Immunoprecipitation was performed by adding 1 µg of the corresponding antibody to 1 mg of lysate. The mixture was gently inverted on a rotator overnight at 4 °C. After the primary antibody incubation, 30 µL of protein A-conjugated magnetic beads (88845, Thermo Fisher, Waltham, MA, USA) were added and incubated for 1 h to bind with the antibody. To isolate the protein–antibody–bead complex from the lysate, the mixture was centrifuged at 3000 rpm for 3 min at 4 °C and washed 4 times with lysis buffer. As a control for the precipitates, 2% of the initial protein amount used for pulldown was prepared as input. The collected complexes and inputs were used for western blots.

### 4.11. Statistics

Statistical analysis was performed using Prism10 (GraphPad Software, La Jolla, CA, USA). Statistical significance was assessed with Tukey’s post hoc test following a one-way analysis of variance (ANOVA). *p*-values less than 0.05 were considered statistically significant. Each error bar represents the SEM for parametric data.

## Data Availability

The data supporting the findings of this study are available upon request from the corresponding author.

## Supplementary Materials

The following supporting information can be downloaded at: www.mdpi.com/article/10.3390/ijms26062649/s1.
